# The association of metabolic syndrome components and diabetes mellitus: evidence from China National Stroke Screening and Prevention Project

**DOI:** 10.1186/s12889-019-6415-z

**Published:** 2019-02-14

**Authors:** Wenzhen Li, Dongming Wang, Xiaojun Wang, Yanhong Gong, Shiyi Cao, Xiaoxv Yin, Xianbo Zhuang, Wenhuan Shi, Zhihong Wang, Zuxun Lu

**Affiliations:** 1 Department of Social Medicine and Health Management, School of Public Health, Tongji Medical College, Huazhong University of Science and Technology, No. 13 Hangkong Road, Wuhan, 430030 China; 2 Department of Occupational & Environmental Health, School of Public Health, Tongji Medical College, Huazhong University of Science and Technology, Wuhan, 430030 China; 3 Department of Neurology, Liaocheng People’s Hospital, Liaocheng city, Shandong Province China; 4 Department of science and education, The Third Hospital of Shijiazhuang, Shijiazhuang city, Hebei Province China; 5 Health Science Center, Shenzhen second people’s hospital, The First Affiliated Hospital of Shenzhen University, Shenzhen, 518020 People’s Republic of China; 6 Shenzhen, China

**Keywords:** Metabolic syndrome, Diabetes mellitus, National cross-sectional study

## Abstract

**Background:**

The metabolic syndrome (MetS) is related with cardiovascular disease. However, its relationship with diabetes mellitus (DM) has not been examined in Chinese population with a larger sample. We aimed to assess the relationship between metabolic syndrome (MetS) and its components, and DM, and to determine the best one from the available definitions of Mets when assessing the risk of DM.

**Methods:**

This was a cross-sectional survey in a nationally representative sample of 109,551 Chinese adults aged ≥40 years in 2014–15. MetS was defined according to three criteria including the updated International Diabetes Federation (IDF) criterion, the National Cholesterol Education Program Third Adult Treatment Panel (NCEP ATP III) criterion and American Heart Association/National Heart, Lung, and Blood Institute (AHA/NHLBI) criterion. Logistic regression models were used to estimate the odds of DM.

**Results:**

MetS as defined by three criteria including IDF, NCEP ATP III,and AHA/NHLBI all increased the prevalence of DM, and the adjusted ORs with 95% CI was more higher using NCEP ATP III (3.65, 3.52–3.79) than IDF (2.50, 2.41–2.60) and AHA/NHLBI (3.03, 2.92–3.24). The odds of DM was highest in hyperglycemia with cut-off glucose≥6.1 mmol/L (14.55, 13.97–15.16), and other components were also associated significantly with DM. There was heterogeneity for OR of DM associated with various trait combinations.

**Conclusions:**

The NCEP ATPIII MetS definition may be more suitable for assessment of DM risk in Chinese population. Hyperglycemia, as previous study reported, are important risk factors of DM. Besides, other traits of Mets are also significantly associated with DM and should therefore be of greater concern.

## Background

The prevalence of diabetes mellitus (DM) for all age-groups worldwide was estimated to be 4.4% (366 million) in 2030, and in China, the total number of people with DM will rise to 42.3 million [1]. Although no cure method was available for diabetes, the disease and its complications could be prevented, delayed and managed by identifying risk factors and detecting the condition at an early stage [2], and numerous clinical trials have shown that metabolic syndrome (MetS), a cluster of risk factors including abdominal obesity, elevated blood pressure, dyslipidemia and dysglycemia [3], is an important risk factor for DM. MetS was a strong predictor of incident DM [4–6], and which was reported to precede the risk of incidence of DM by as much as 5 years [7]. However, the clinical application and practicality of MetS remained debated [8, 9].

Although many reports have examined the relationship between MetS and incident DM, there still has been much controversy with regard to certainty of the definition and its value to identify persons with a high risk of DM [10]. It remains an issue whether different definitions of MetS yield similar estimates for risk of DM. Previous study suggested that the risk of DM associated with MetS depended both upon the used definition and the studied population [11]. However, large-scale epidemiology studies were scarce in China [12].

As we all know, obesity and impaired glucose which are included in definition of MetS are two major risk factors for DM [13], and therefore, the MetS should be a risk factor for diabetes. But, there is an ongoing debate as to whether the predictive value of the MetS is mainly attributable to the impaired glucose regulation component. The American Diabetes Association and the European Association for the Study of Diabetes suggested that the association between MetS and DM may due to impaired fasting glucose or impaired glucose tolerance [10]. However, the results from the Framingham Study indicated that trait combinations that did not include fasting glucose also imparted an increased risk for incident DM [14]. Thus, it is necessary to separate the effect of each component of MetS, which is likely to benefit from early intervention strategies.

Thus, we conducted a large-scale epidemiological study in a middle-aged and elderly Chinese population. The objective of this study was to compare the effects of different MetS definitions, including updated International Diabetes Federation (IDF) definition [15], the National Cholesterol Education Program Third Adult Treatment Panel (NCEP ATP III) definition [16] and American Heart Association/National Heart, Lung, and Blood Institute (AHA/NHLBI) definition [17], as well as various components on DM in a Chinese middle-aged and elderly population. Meanwhile, we aimed to explore a more reasonable definition of Mets when analyzing the risk of DM in Chinese population.

## Methods

### Study subjects and design

This data was from China National Stroke Screening and Prevention Project, which was a community-based cross-sectional study that was administrated by the National Project Office of Stroke Prevention and Control and carried out in 30 provinces in mainland China from October 2014 to November 2015. A 2-stage stratified cluster sampling method was used, and 200 project areas were determined firstly in proportion to the local population size and numbers of total counties and in each project area, an urban community and a rural village were selected as primary sampling units according to the geographical environment and suggestions from local hospitals. Cluster sampling method was used in every primary sampling unit and all residents aged ≥40 years were surveyed in primary screening process. Questionnaire survey, physical examination, and risk factors of stroke assessment were conducted in primary health care institutions. A sample of study participants was randomly selected in each primary sampling unit for further survey in laboratory tests, carotid ultrasound, and electrocardiogram. Nevertheless, participants were excluded if they had severe illnesses, and were unable to attend questionnaire survey or physical examination. The present analysis was restricted to individuals who had completed all surveys.

### Definition of metabolic syndrome

An individual was considered to have MetS by the updated IDF definition if he or she fitted the following criteria:

Central obesity (waist circumference ≥ 90 cm in male and ≥ 80 cm in female) plus any two of four additional factors: (1) hypertriglyceridemia: Triglyceride (TG) level ≥ 1.7 mmol/L; (2) high blood pressure: blood pressure ≥ 130/85 mmHg or treatment of previously diagnosed hypertension; (3) reduced high-density lipoprotein (HDL)-cholesterol: < 1.03 mmol/L in males and 1.29 mmol/L in females or specific treatment for these lipid abnormalities; (4) hyperglycemia: fasting glucose level of ≥5.6 mmol/L or treatment of previously diagnosed type 2 diabetes.

An individual was considered to have MetS by the NCEP ATP IIIdefinition if he or she has three or more of the following criteria:

(1) Central obesity: waist circumference ≥ 102 cm in male and ≥ 88 cm in female; (2) high blood pressure: blood pressure ≥ 130/85 mmHg or treatment of previously diagnosed hypertension; (3) reduced HDL cholesterol were the same as those of the IDF; (4) hypertriglyceridemia: TG level ≥ 1.7 mmol/L; (5) hyperglycemia: fasting glucose level of ≥6.1 mmol/L or treatment of previously diagnosed type 2 diabetes.

An individual was considered to have MetS by the AHA/NHLBI definition if he or she has three or more of the following criteria:

(1) Central obesity: waist circumference ≥ 90 cm in male and ≥ 80 cm in female; (2) high blood pressure: blood pressure ≥ 130/85 mmHg or treatment of previously diagnosed hypertension; (3) reduced HDL cholesterol were the same as those of the IDF; (4) hypertriglyceridemia: TG level ≥ 1.7 mmol/L; (5) hyperglycemia: fasting glucose level of ≥5.6 mmol/L or treatment of previously diagnosed type 2 diabetes.

### Ascertainment of DM

DM was assessed according to the diagnosis of physicians. Individuals were defined as DM if they met one of the following criteria: (1) self-reported physician diagnosis of DM; (2) self-reported current use of anti-diabetic medication or insulin.

### Covariates

Information on sociodemographic characteristics (age, gender, education, and marital status), lifestyle, chronic diseases history, and physical activity were collected through a questionnaire by face to face interview with trained interviewers. Individuals smoking at least one cigarette per day for more than half a year were defined as current smokers, and those who drink at least one time per week for more than half a year were defined as current drinkers. Physical activity was defined as regularly exercised more than 3 times/weeks and ≥ 30 min/times or engaged in heavy physical work. Family history of DM diagnosed by physicians was reported by the participants. Body mass index, waistline, systolic blood pressure (SBP), diastolic blood pressure (DBP), triglycerides, total cholesterol, high density lipoproteincholesterol (HDL-C), low density lipoproteincholesterol (LDL –C), and fasting glucose were measured using standard procedures.

### Data analysis

The t test and Chi-square test were used to examine differences in continuous and categorical variables between males and females respectively. Logistic regressions were used to calculate odds ratios (ORs) and 95% confidence intervals (CIs), including the association of different definitions of Mets and DM, and the relationship of various components of Mets and DM. We chose covariates according to evidence from published literatures [18, 19]. Potential confounders included age, gender, marital status, level of education, current smoking, current drinking, physical activity, and family history of diabetes mellitus. All analyses were conducted using SPSS 19.0 (SPSS Inc., Chicago, Ill). The statistical tests were two sided, and significance was set at *P <* 0.05.

## Results

The present study included 109,551participants. Table 1 showed the basic characteristics of the 109,551 subjects (49,789 men and 59,762 women). Among them, a total of 15,799 individuals had diabetes mellitus, including 6799 males (13.7%) and 9000 females (15.1%). Small but statistically significant differences were found between men and women in other demographic, anthropometric and clinical characteristics. Interesting, the difference was significant in DM (*P* < 0.001), but not for BMI (*P* = 0.306) and fasting glucose (*P* = 0.292).

**Table 1:** Demographic, Anthropometric, and Plasma Biochemical Characteristics of Subjects

Characteristic	Male(*n* = 49,789)	Female(*n* = 59,762)	*P* Value
Mean age(mean, SD),years	59.15 ± 11.150	59.30 ± 10.894	0.028
Age, years			
40~49	11,842 (23.8)	13,095 (21.9)	< 0.001
50~59	13,872 (27.9)	17,504 (29.3)	
≥ 60	24,075 (48.4)	29,163 (48.8)	
Education			
Primary school and below	17,504 (35.2)	28,786 (8.2)	< 0.001
Middle school	19,277 (38.7)	19,018 (31.8)	
High school or the equivalent	8339 (16.7)	8623 (14.4)	
University or other tertiary degree	4669 (9.4)	3335 (5.6)	
Marital status			
Married	47,467 (95.3)	54,729 (91.6)	< 0.001
Single or divorced	1001 (2.0)	844 (1.4)	
Widowed	1321 (2.7)	4189 (7.0)	
Physical activity^a^	37,637 (75.6)	44,241 (74.0)	< 0.001
Smokers	21,378 (42.9)	2196 (3.7)	< 0.001
Drinkers	13,581 (27.3)	1485 (2.5)	< 0.001
Diabetes mellitus	6799 (13.7)	9000 (15.1)	< 0.001
Family history of diabetes mellitus	2904 (5.8)	3839 (6.4)	< 0.001
Anti-diabetic medication in two weeks	4282 (8.6)	5663 (9.5)	< 0.001
Body mass index(mean, SD)	24.87 ± 3.520	24.90 ± 3.809	0.306
Waistline (cm) (mean, SD)	87.55 ± 10.45	84.19 ± 10.45	< 0.001
Waist circumference(cm)(NCEP ATPIII and AHA/NHLBI)^b^	3608 (7.2)	20,215 (33.8)	< 0.001
Waist circumference(cm)(IDF)^c^	19,964 (40.1)	40,999 (68.6)	< 0.001
Systolic blood pressure(mmHg)	134.94 ± 18.47	134.00 ± 19.85	< 0.001
Diastolic blood pressure(mmHg)	83.23 ± 11.30	81.44 ± 11.21	< 0.001
Triglycerides(mmol/l)	1.76 ± 1.36	1.73 ± 1.18	0.003
Total cholesterol(mmol/l)	4.77 ± 1.09	5.01 ± 1.15	< 0.001
HDL cholesterol(mmol/l)	1.43 ± 0.61	1.50 ± 0.57	< 0.001
LDL cholesterol(mmol/l)	2.80 ± 0.93	2.92 ± 0.97	< 0.001
Fasting glucose(mmol/L)	5.76 ± 1.76	5.75 ± 1.77	0.292
Fasting glucose(mmol/l)(NCEP ATP III)^d^	11,353 (22.8)	13,242 (22.2)	0.011
Fasting glucose(mmol/l)(AHA/NHLBI and IDF)^e^	19,846 (39.9)	23,158 (38.8)	< 0.001

^a^Times of physical activity≥3/weeks and ≥ 30 min/times or the person is an industrial/argricular labor; ^b^waist circumference ≥ 102 cm in male and ≥ 88 cm in female; ^c^ waist circumference ≥ 90 cm in male and ≥ 80 cm in female; ^d^fasting glucose level of ≥5.6 mmol/L; ^e^fasting glucose level of ≥6.1 mmol/L

*SD* standard deviation, *NCEP ATPIII* National Cholesterol Education Program Third Adult Treatment Panel, *AHA/NHLBI* American Heart Association/National Heart, Lung, and Blood Institute, *IDF* International Diabetes Federation, *HDL* high-density lipoprotein, *LDL*-low density lipoprotein

Table 2 showed the estimated association of DM and MetS or components of MetS by different MetS criteria. The MetS was significantly associated with DM and the ORs of the disease associated with MetS defined by ATP was greater (OR = 3.65, 95% CI = 3.52–3.79) than the other two criteria with the adjustment of covariates (OR = 3.03, 95% CI = 2.92–3.14 for AHA/NHLBI, OR = 2.50, 95% CI = 2.41–2.60 for IDF). When the MetS criteria were combined, it indicated that the odds of DM was highest based on MetS by AHA/NHLBI and NCEP ATP III (OR = 3.65, 95% CI = 3.52–3.79), compared with non- MetS individuals when adjusting for other variables. As to the MetS components, it revealed that the number of MetS components was related with DM in a dose-response way, regardless of different definitions of MetS. And five components of MetS by NCEP ATP III got the highest risk (OR = 26.40, 95% CI = 22.90–30.43).

**Table 2:** Odds and 95% Confidence Interval (CI) of Diabetes Mellitus for MetS and the Number of Components of MetS by the NCEP, AHA and IDF Criteria

	DM(*n* = 15,799)	
	Crude OR(95% CI)	AdjOR(95% CI)
MetS by different criteria(Ref. = Non-MetS)		
NCEP ATP III	4.12 (3.98–4.26)	3.65 (3.52–3.79)
AHA/NHLBI	3.46 (3.34–3.58)	3.03 (2.92–3.14)
IDF	2.84 (2.74–2.93)	2.50 (2.41–2.60)
Subgroup of MetS(Ref. = Non-MetS)		
MetS by IDF and NCEP ATP III	4.96 (4.77–5.17)	3.06 (2.94–3.18)
MetS by IDF and AHA/NHLBI	4.33 (4.16–4.51)	2.63 (2.53–2.73)
MetS by AHA/NHLBI and NCEP ATP III	4.11 (3.97–4.26)	3.65 (3.52–3.79)
Nunber of components of MetS by NCEP ATP III(Ref. = zero)		
One	2.08 (1.87–2.32)	1.78 (1.59–1.98)
Two	5.24 (4.74–5.81)	4.35 (3.93–4.83)
Three	9.84 (8.88–10.91)	7.86 (7.08–8.72)
Four	18.24 (16.38–30.31)	13.93 (12.47–15.56)
Five	34.42 (30.03–39.46)	26.40 (22.90–30.43)
*P for trend*		< 0.001
Nunber of components of MetS by AHA/NHLBI(Ref. = zero)		
One	2.48 (2.19–2.82)	2.11 (1.86–2.40)
Two	6.12 (5.42–6.90)	4.93 (4.36–5.57)
Three	10.60 (9.39–11.97)	8.17 (7.23–9.24)
Four	17.19 (15.18–19.46)	12.60 (11.10–14.30)
Five	26.18 (22.70–30.20)	19.10 (16.41–22.03)
*P for trend*		< 0.001
Nunber of components of MetS by IDF(Ref. = zero)		
One	2.28 (1.95–2.67)	2.01 (1.72–2.36)
Two	4.70 (4.308–5.46)	3.88 (3.33–4.52)
Three	9.432 (8.13–10.95)	7.406.36–8.61)
Four	14.65 (12.60–17.03)	11.06 (9.49–12.88)
Five	26.57 (22.69–31.12)	18.81 (16.01–22.10)
*P for trend*		< 0.001

Adjusted for sex, age (40~49, 50~59, ≥60), marital status, education, physical activity (3 times/weeks and ≥ 30 min/times or is industrial and agricular labor), current smoking (yes, no), current drinking (yes, no), family history of diabetes mellitus

*MetS* metabolic syndrome, *NCEP ATPIII* National Cholesterol Education Program Third Adult Treatment Panel, *AHA/NHLBI* American Heart Association/National Heart, Lung, and Blood Institute, *IDF* International Diabetes Federation, *HDL* high-density lipoprotein, *Ref* reference

For NCEP ATP III criterion, component one to five are one or more of five components including central obesity (waist≥102/88), elevated BP, hypertriglyceridemia, low HDL and hyperglycemia (glucose≥6.1); For AHA/NHLBI criterion, component one to five are one or more of five components including central obesity (waist≥102/88), elevated BP, hypertriglyceridemia, low HDL and hyperglycemia (glucose≥5.6); For IDF criterion, component one to five are one or more of five components including central obesity (waist≥90/80), hypertriglyceridemia, elevated BP, low HDL and hyperglycemia (glucose≥5.6)

Table 3 showed the effects of various combinations of MetS traits on DM according to the presence of single component and their combination in pairs, triplets and quartets. The OR of DM associated with specific trait combinations was estimated with the group without that specific combination used as the comparator. The analysis suggested that heterogeneity for DM associated with various trait combinations. In terms of single component, hyperglycemia (glucose≥6.1) was associated with the highest prevalence of DM (OR = 14.55, 95% CI = 13.97–15.16), two components (hyperglycemia (glucose≥6.1) and elevated BP) was associated with the highest prevalence of DM (OR = 9.90, 95% CI = 9.50–10.30), three components (hyperglycemia (glucose≥6.1), central obesity (waist≥90/80) and elevated BP) was associated with the highest prevalence of DM (OR = 7.45, 95% CI = 7.14–7.78), four components (hyperglycemia (glucose≥6.1), hypertriglyceridemia, central obesity (waist≥90/80) and elevated BP) was associated with the highest prevalence of DM (OR = 5.83, 95% CI = 5.52–6.15). Besides, the analyses based on different combinations indicated that the OR of DM was higher in the combination including hyperglycemia (glucose≥6.1) than other combinations.

**Table 3:** Crude Odds^a^ and multi-adjusted odds^a^ and 95% Confidence Interval (CI) of Diabetes Mellitus for the Specific Combination of MetS Traits Relative to Individual Without That Combination

Model	MetS Traits							No. of Subjects (%)	DM	
	Hyperglycemia (glucose≥5.6)	Hyperglycemia (glucose≥6.1)	Hypertrigly-ceridemia	Central obesity (Waist≥90/80)	Central obesity (Waist ≥102/88)	Elevated BP	Low HDL		Crude OR (95% CI)	Adj-OR (95% CI)
1. MetS component										
1.1	*							43,004 (39.3)	10.28 (9.84–10.74)	8.81 (8.43–9.21)
1.2		*						24,595 (22.5)	17.04 (16.38–17.73)	14.55 (13.97–15.16)
1.3			*					43,572 (39.8)	1.34 (1.29–1.38)	1.34 (1.29–1.39)
1.4				*				60,963 (55.6)	1.67 (1.61–1.73)	1.50 (1.45–1.56)
1.5					*			23,823 (21.7)	1.75 (1.69–1.82)	1.55 (1.49–1.62)
1.6						*		72,474 (66.2)	2.08 (2.00–2.16)	1.62 (1.55–1.69)
1.7							*	33,471 (30.6)	1.28 (1.24–1.33)	1.27 (1.23–1.32)
2. MetS component										
2.1	*		*					19,209 (17.5)	4.08 (3.93–4.24)	3.65 (3.51–3.79)
2.2	*			*				26,499 (24.2)	5.38 (5.20–5.58)	4.65 (4.48–4.83)
2.3	*				*			11,586 (10.6)	4.09 (3.92–4.26)	3.59 (3.43–3.76)
2.4	*					*		32,001 (29.2)	6.39 (6.16–6.63)	5.33 (5.14–5.54)
2.5	*						*	13,658 (12.5)	4.05 (3.89–4.22)	3.63 (3.48–3.79)
2.6		*	*					11,874 (10.8)	7.78 (7.46–8.11)	6.80 (6.51–7.11)
2.7		*		*				15,988 (14.6)	10.12 (9.74–10.52)	8.66 (8.31–9.02)
2.8		*			*			7373 (6.7)	7.36 (7.00–7.73)	6.47 (6.13–6.83)
2.9		*				*		19,161 (17.5)	11.77 (11.33–12.22)	9.90 (9.52–10.30)
2.10		*					*	8294 (7.6)	8.03 (7.66–8.42)	7.00 (6.66–7.36)
2.11			*	*				26,102 (23.8)	1.65 (1.59–1.72)	1.53 (1.47–1.59)
2.12			*		*			10,723 (9.8)	1.96 (1.86–2.05)	1.69 (1.60–1.78)
2.13			*			*		29,425 (26.9)	1.74 (1.68–1.80)	1.55 (1.50–1.61)
2.14			*				*	18,484 (16.9)	1.36 (1.30–1.42)	1.34 (1.28–1.40)
2.15				*		*		44,128 (40.3)	1.90 (1.83–1.96)	1.57 (1.51–1.63)
2.16				*			*	21,208 (19.4)	1.55 (1.49–1.61)	1.45 (1.39–1.52)
2.17					*	*		18,652 (17.0)	1.92 (1.85–2.00)	1.62 (1.55–1.69)
2.18					*		*	9175 (8.4)	1.84 (1.74–1.93)	1.62 (1.53–1.71)
2.19						*	*	21,337 (19.5)	1.71 (1.64–1.77)	1.50 (1.44–1.57)
3. MetS component										
3.1	*		*	*				12,730 (11.6)	4.04 (3.88–4.21)	3.49 (3.34–3.65)
3.2	*		*		*			5850 (5.3)	3.99 (3.77–4.22)	3.35 (3.16–3.56)
3.3	*		*			*		14,523 (13.3)	4.19 (4.03–4.37)	3.57 (3.43–3.73)
3.4	*		*				*	8152 (7.4)	3.70 (3.52–3.88)	3.45 (3.08–3.42)
3.5	*			*		*		20,770 (19.0)	4.91 (4.74–5.09)	4.07 (3.91–4.23)
3.6	*			*			*	9525 (8.7)	4.14 (3.96–4.34)	3.60 (3.43–3.79)
3.7	*				*	*		9486 (8.7)	4.16 (3.97–4.35)	3.51 (3.34–3.69)
3.8	*				*		*	4664 (4.3)	4.05 (3.81–4.31)	3.44 (3.22–3.67)
3.9		*	*	*				8218 (7.5)	7.21 (6.88–7.56)	6.15 (5.85–6.47)
3.10		*	*		*			3978 (3.6)	6.70 (6.28–7.14)	5.62 (5.25–6.03)
3.11		*	*			*		9405 (8.6)	7.48 (7.15–7.82)	6.37 (6.07–6.67)
3.12		*	*				*	5194 (4.7)	6.84 (6.46–7.25)	5.84 (5.50–6.21)
3.13		*		*		*		12,920 (11.8)	8.97 (8.61–9.34)	7.45 (7.14–7.78)
3.14		*		*			*	6029 (5.5)	7.68 (7.28–8.10)	6.51 (6.15–6.90)
3.15		*			*	*		6176 (5.6)	7.26 (6.89–7.66)	6.18 (5.83–6.54)
3.16		*			*		*	3096 (2.8)	7.05 (6.55–7.76)	5.97 (5.52–6.46)
3.17			*	*		*		19,335 (17.6)	1.87 (1.80–1.95)	1.62 (1.56–1.69)
3.18			*	*			*	12,056 (11.0)	1.63 (1.55–1.71)	1.51 (1.43–1.59)
3.19			*		*	*		8608 (7.9)	2.09 (1.99–2.21)	1.74 (1.64–1.84)
3.20			*		*		*	5282 (4.8)	1.99 (1.86–2.12)	1.72 (1.60–1.84)
3.21				*		*	*	14,726 (13.4)	1.87 (1.79–1.96)	1.62 (1.54–1.69)
3.22					*	*	*	7037 (6.4)	2.06 (1.95–2.18)	1.74 (1.64–1.85)
4. MetS component										
4.1	*		*	*		*		10,101 (9.2)	4.08 (3.91–4.27)	3.41 (3.25–3.58)
4.2	*		*	*			*	5809 (5.3)	3.94 (3.72–4.17)	3.32 (3.13–3.53)
4.3	*		*		*	*		4854 (4.4)	4.08 (3.84–4.34)	3.34 (3.13–3.56)
4.4	*		*		*		*	2889 (2.6)	4.06 (3.76–4.38)	3.36 (3.09–3.65)
4.5		*	*	*		*		6720 (6.1)	6.98 (6.63–7.35)	5.83 (5.52–6.15)
4.6		*	*	*			*	3840 (3.5)	6.88 (6.44–7.34)	5.67 (5.29–6.08)
4.7		*	*		*	*		3370 (3.1)	6.67 (6.22–7.15)	5.49 (5.10–5.92)
4.8		*	*		*		*	2003 (1.8)	6.76 (6.15–7.39)	5.58 (5.08–6.14)
4.9			*	*		*	*	8593 (7.8)	1.95 (1.85–2.06)	1.68 (1.59–1.78)
4.1			*		*	*	*	4147 (3.8)	2.20 (2.05–2.36)	1.83 (1.70–1.98)

^a^The ORs are shown for person with the metabolic syndrome trait combination specific compared with persons without that combination

Adjusted for sex, age (40~49, 50~59, ≥60), marital status, education, physical activity (3 times/weeks and ≥ 30 min/times or is industrial and agricular labor), current smoking (yes, no), current drinking (yes, no), family history of DM

*DM* diabetes mellitus, *MetS* Metabolic syndrome

## Discussions

In the present study, MetS as defined by three criteria all increased the prevalence of DM in Chinese middle-aged and elderly population, thus it verified that MetS was an important risk factor for DM among Chinese population. However, the three MetS definition did not seem to yield the same estimates for risk of DM and the NCEP ATP III MetS definition may be more suitable for assessment of DM risk with the highest increased risk of DM. To our knowledge, it is the first study to compare different definitions and components of MetS when assessing its relationship with DM in a large Chinese population. The results of our study could provide a reference when the MetS definition was conducted among Chinese adults.

Our study also showed that hyperglycemia was significantly associated with an increased risk of DM after adjusting for sex, age, marital status, education, physical activity, current smoking, current drinking, and family history of DM. Previous studies suggested impaired fasting glucose (IFG) was the strongest predictor of type 2 diabetes among the components [20–22], which was also confirmed in our study, the hyperglycemia with cut-off ≥6.1 mmol/L was associated with the highest prevalence of DM in all components. Besides, hypertriglyceridemia was also associated with increased risk of DM after adjusting confounding factors in the present study. Raised plasma triglyceride is thought to play a role in the pathogenesis of insulin-resistant diabetes and may be used as a biomarker for the prediction of risk of type 2 diabetes [23]. Some experimental studies [24] and human case reports also supported that hypertriglyceridemia could predict insulin resistance and glucose intolerance and showed that insulin-resistant diabetes could be caused by extremely high levels of triglycerides [25].

Central obesity, defined as an essential component in some MetS diagnostic criteria, such as those of the IDF [3] or the Japanese criteria [26], has been reported to be an important factor in the development of DM [27]. Besides, our results also showed central obesity was not the indispensable component of MetS for the risk of DM. Other components such as elevated BP and low HDL cholesterol yield similar effect on prevalence of DM, and a previous studies have shown that elevated BP and low HDL cholesterol were associated with insulin resistance [28] and therefore may contributed to the occurrence of DM. The results also indicated the importance of blood pressure in the prevention and management of DM [29, 30]. It was consistent with a previous longitudinal study [31], which showed the incidence of T2DM was higher among individuals with higher BP and low HDL cholesterol level. However, no causal association between low HDL cholesterol levels and T2DM was found in another prospective study [32]. Our results supported that the MetS, as a risk factor of DM, should be considered as a clinical entity independent of prediabetes or DM [33].

Epidemiological studies revealed that the risk of multiple risk factors was much more than the sum of accompanying single risk factors [34], and whether the risk of MetS was greater than the sum of its individual components that came up in cardiovascular field has been debated [35]. Thus, we further explored the relationship between different trait combinations and risk of DM, and we found that there were differences of associations of trait combinations of MetS with DM, which may result from the different pathological mechanisms and the interactive effects of components. A study with 1844 subjects showed IFG added with two or more other components apparently increased the risk of DM compared to IFG alone [36], but the effects did not seem to be observed in our results. The differences in study design, study population, study sample and adjusted confounders, etc. may contribute to the different results. Further studies with larger sample and longer follow-up time cohort study should be conducted to examine the results.

With the aging, industrialization, economic transition and sedentary lifestyle, there was a striking increase in the number of individuals with MetS in China [37]. A survey [38] performed in 97,098 Chinese residents from 31 provinces showed the prevalence of the MetS was 33.9%, which will result in the increased risk of DM inevitably due to significant association of them, therefore, developing effective public health strategies for the control and treatment of MetS should be an urgent priority to reduce the social and medical burden of DM in China.

Some limitations of the study are worth mentioning. First of all, the study is a cross-sectional study and limits to confirm the causal inference, which is a main limitation in epidemiological studied in general. Secondly, DM and other confounders were self-reported, which may have led to misclassification and underestimated or over-estimated the association of MetS with the risk of DM. In addition, although our analyses were adjusted for smoking and alcohol consumption, residual confounders remain due to lifestyle factors could not be excluded. Thirdly, the measured glucose was just fasting glucose, not the 2-h post-meal glucose or HbA1c, which may influence the results. Fourthly, communities and villages were selected as primary sampling units according to suggestions from local hospitals in the present study, although it was a practical and more reliable approach, subjective problem may be not excluded. Last but not least, we could not distinguish the type 1 DM and type 2 DM due to data limitation, which should be explored in the future study.

## Conclusions

Our study suggests that the MetS defined by three criteria were associated with DM, and the NCEP ATP III MetS definition may be more suitable for assessment of DM risk than other two definitions in Chinese population. Besides, other traits of MetS and their combinations are also significantly associated with DM and should therefore be more concerned as soon as possible so as to change the growing trend of MetS and DM in China. Further studies with all ages and different ethnic groups should be conducted in the future.

## Abbreviations

AHA/NHLBI: American Heart Association/National Heart, Lung, and Blood Institute
CI: Confidence interval
DBP: Diastolic blood pressure
DM: Diabetes mellitus
HDL: High-density lipoprotein
HDL-C: Lipoproteincholesterol
IDF: International Diabetes Federation
LDL –C: Low density lipoproteincholesterol
MetS: Metabolic syndrome
NCEP ATP III: National Cholesterol Education Program Third Adult Treatment Panel
OR: Odds ratio
SBP: Systolic blood pressure
TG: Triglyceride
